# Immunoglobulins G from Patients with Systemic Sclerosis Modify the Molecular Signatures of Endothelial Cells

**DOI:** 10.1136/rmdopen-2024-004290

**Published:** 2025-03-23

**Authors:** Aurélien Chepy, Solange Vivier, Fabrice Bray, Clément Chauvet, Alain Lescoat, Abderrahmane Elhannani, Martin Figeac, Lucile Guilbert, Frédéric Leprêtre, Louisa Bourel, Eric Hachulla, Christian Rolando, Valérie Lecureur, Sylvain Dubucquoi, David Launay, Vincent Sobanski

**Affiliations:** 1 Univ. Lille, Inserm, CHU Lille, U1286 - INFINITE - Institute for Translational Research in Inflammation, Lille, France; 2 CHU Lille, Département de Médecine Interne et Immunologie Clinique, Centre de Référence des Maladies Auto-Immunes et Auto-Inflammatoires Rares du Nord, Nord-Ouest, Méditerranée et Guadeloupe (CeRAINOM), Lille, France; 3 Univ. Lille, CNRS, USR 3290 - MSAP - Miniaturisation pour la Synthèse, l'Analyse et la Protéomique, Lille, France; 4 Service de Médecine Interne et Immunologie Clinique, CHU Rennes, Université Rennes, Rennes, France; 5 Inserm, EHESP, Irset (Institut de Recherche en Santé, Environnement et Travail) - UMR_S 1085, Rennes, France; 6 Univ. Lille, CNRS, Inserm, CHU Lille, Institut Pasteur de Lille, US 41 - UAR 2014 - PLBS, Lille, France; 7 CHU Lille, Institut d’Immunologie, Lille, France; 8 Institut Universitaire de France (IUF), Paris, France

**Keywords:** Scleroderma, Systemic, Autoantibodies, Autoimmune Diseases

## Abstract

**Objective:**

Antinuclear antibodies (ANA) are powerful biomarkers in systemic sclerosis (SSc). Functional antibodies (FA) might be implicated in vasculopathy, in which endothelial cells (EC) are key players. We aimed to explore the effect of purified IgG from patients with SSc on omics signatures of EC and examine the influence of ANA serotypes and FA.

**Methods:**

EC were cultured in the presence of purified IgG from patients with SSc, patients with systemic lupus erythematosus (SLE) or healthy controls (HC). EC omics profiles were analysed by liquid chromatography with tandem mass spectrometry (LC-MS/MS) and RNA sequencing. EC proteome induced by IgG from patients with SSc was confirmed with an external validation cohort.

**Results:**

In the derivation cohort, principal component analysis (PCA) using proteomics data showed three distinct groups of subjects: a first one including mostly anti-topoisomerase-I positive patients (ATA+), a second one including mostly anti-centromere positive patients and a third group comprising anti-RNA polymerase-III positive patients, SLE and HC. In transcriptomics, PCA distinguished one group composed of ATA+patients only from a second group mixing ATA+patients with other individuals. The validation cohort confirmed the existence of two groups of distinct EC proteome profiles and clinical severity in ATA+patients. In both SSc cohorts, no association between FA presence and proteomic profiles was observed. Quantitative proteomics measured the most discriminant proteins in EC exposed to purified IgG.

**Conclusion:**

Purified IgG from patients with SSc can modify EC proteome and transcriptome. The observed changes closely associate with ANA serotype.

**WHAT IS ALREADY KNOWN ON THIS TOPIC**

- Antinuclear antibodies (ANA) are strong biomarkers in systemic sclerosis (SSc) but their implication in pathophysiology remains unclear.
- Functional antibodies (FA) might contribute to SSc pathogenesis.

**WHAT THIS STUDY ADDS**

- Purified IgG from patients with SSc can modify endothelial cells (EC) proteome and transcriptome according to ANA serotypes with a minimal implication of FA.
- Some anti-topoisomerase-I positive (ATA+) patients induced singular and distinct molecular EC profiles which were not explained by FA.
- The ATA+group comprised two subgroups which differed in terms of proteomic profiles and organs involvement.

**HOW THIS STUDY MIGHT AFFECT RESEARCH, PRACTICE OR POLICY**

- IgG could be pathogenic, reinforcing the need for therapeutic targeting of humoral immunity, particularly in ATA+patients.
- The EC molecular signature induced by IgG from patients with SSc could help uncover patient differences within the same ANA serotype.

## Introduction

 Systemic sclerosis (SSc) is a heterogeneous connective tissue disease characterised by autoimmunity, vasculopathy and excessive fibrosis.^1^ Skin fibrosis defines the disease in two forms according to its extent: limited cutaneous SSc (lcSSc), in which skin involvement does not extend beyond the elbows and knees and diffuse cutaneous SSc (dcSSc), in which skin involvement is more extensive. In some cases, fibrosis can extend to internal organs, causing severe complications such as interstitial lung disease (ILD). Vasculopathy is present in most patients, varying from Raynaud’s phenomenon and telangiectasia to pulmonary arterial hypertension (PAH) or renal crisis.^1^ The humoral immune system plays an important role in SSc and autoantibodies (Aab) are found in almost patients with SSc.^2^ ‘Functional antibodies’ (FA) such as angiotensin II type 1 receptor (AT1R) Aab and endothelin-1 type A receptor (ETAR) Aab have been described and might be implicated in the vasculopathy of SSc, in which endothelial cells (EC) are key players.^3^ Although FA are not used in clinical daily practice, antinuclear antibodies (ANA) are powerful diagnosis and prognosis biomarkers. Three of them are part of the last American College of Rheumatology (ACR)/European Alliance of Associations for Rheumatology (EULAR) SSc classification criteria: anti-topoisomerase-I (ATA) usually associated with dcSSc and ILD, anti-centromere (ACA) with lcSSc and PAH and anti-RNA polymerase-III Aab (ARA) with dcSSc and renal crisis.^4^ The role of ANA in the pathogenicity of SSc is still partially unknown. Omics studies have proven valuable for investigating the effects of Aab on target cells. A previous study from our group showed that IgG from patients with SSc could alter the proteome and transcriptome of fibroblasts, highlighting a unique molecular profile induced by IgG from ATA-positive (ATA+) patients.^5^

In this study, we investigated whether purified IgG from patients with SSc affects the proteome and transcriptome of EC. We assessed the possible contribution of ANA serotypes and FA on molecular profiles. With a validation cohort, we confirmed a specific profile induced by IgG from ATA+patients.

## Material and methods

### Individuals

Sera from patients (SSc and systemic lupus erythematosus (SLE)) were obtained from our biobanks and used as a derivation cohort (Lille, ethical approval: CPP N°: 2019-A01083-54) or validation cohort (Rennes, CPP N°: 2019-A02611-56). Healthy controls (HC) were obtained from the Etablissement Français du Sang.

### IgG purification

Total IgG were purified from patients or HC sera by affinity chromatography on an ÄKTA-start FPLC system using HiTrap Protein G columns (Cytiva, Marlborough, Massachusetts, USA). Total IgG-level measurement and serotype assay by BioPlex ANA screen (BioPlex 2200 ANA Screen (Bio-Rad Laboratories, Hercules, California, USA, according to the manufacturer’s instructions) were performed to ensure the validity of the purification step (for both derivation and validation cohort).

### Anti-AT1R and anti-ETAR ELISA

AT1R and ETAR Aab were quantified and analysed with ELISA kits according to the manufacturer’s instructions (CellTend, Luckenwalde, Germany). Briefly, the concentration of each purified IgG was adjusted to 10 µg/µL with phosphate-buffered saline (Gibco, Thermo Fisher Scientific, Waltham, Massachusetts, USA), and samples were diluted in diluent buffer at 1:100. To calculate the antibody concentration, a four-parameter curve was generated under standard conditions. Samples were considered positive if the concentration was >17 U/mL and negative if <10 U/mL.

### Cell culture

Human umbilical vein EC (CC-2519, Lonza, Basel, Switzerland) were cultured in M199 culture medium (Thermo Fisher Scientific, Waltham, Massachusetts, USA) supplemented with 20% fetal calf serum (SVF, Lot RH20220001, Cytiva, Marlborough, Massachusetts, USA) and 10% EBM-2 medium supplemented with EGM-2 SingleQuots kit (endothelial growth media, Lonza, Basel, Switzerland) and used at fifth passage. For derivation cohort, EC were incubated with purified IgG from 10 ATA+patients, 10 ACA+patients, 10 ARA+patients, 10 HC and 5 patients with SLE (all of them positive for anti-DNA antibodies). Three positive controls were set in quadruplicate: EC stimulated by interleukin-1 beta (IL-1β, 2 ng/mL, 240-B, R&D systems, Minneapolis, Minnesota, USA), tumour necrosis factor alpha (TNF-α, 2 ng/mL, 210-TA, R&D systems, Minneapolis, Minnesota, USA) or lipopolysaccharide (LPS, *Escherichia coli* O127:B8, Sigma-Aldrich, Saint-Louis, Missouri, USA); the negative control was constituted by non-stimulated EC (pentaplicate). For validation cohort, EC were incubated with purified IgG from 24 ATA+patients, 18 ACA+patients, 5 ARA+patients and 20 HC. After 24 hours of incubation, the cells were lysed and stored at −80°C until analysis.

For validation cohort, EC were incubated with purified IgG from 24 ATA+patients, 18 ACA+patients, 5 ARA+patients and 20 HC.After 24 hours of incubation, the cells were lysed and stored at −80°C until analysis.

### Sample preparation and liquid chromatography with tandem mass spectrometry

Protein digestion, liquid chromatography with tandem mass spectrometry (LC-MS/MS) analysis and raw data processing were performed as previously described.^5^ For each sample, 1 µg of digested peptide was analysed on a U3000 RSLC Microfluidic HPLC System and an Orbitrap Q-Exactive plus Mass Spectrometer (Thermo Fisher Scientific, Waltham, Massachusetts, USA).

### 3’ messenger RNA sequencing

Total RNA was extracted using the NucleoSpin RNA/Protein kit according to manufacturer’s instructions (Macherey-Nagel, Düren, Germany). Total RNA yield and quality were assessed on the Agilent 2100 Bioanalyzer (Agilent Technologies, Santa Clara, California, USA). Libraries were generated using the QuantSeq 3’ mRNA-Seq Library Prep Kit FWD (Lexogen, Vienna, Austria), pooled equimolarly and sequenced on a NovaSeq 6000 (Illumina, San Diego, California, USA). Reads were aligned with the STAR program (V.2.6.0a) with the genome reference human (GRCh38) and the reference gene annotations (Ensembl) and counted with featureCounts (V.1.6.0).

### Absolute quantification proteomics

Heavy peptide absolute quantification (AQUA) were purchased from Thermo Fisher Scientific (Waltham, Massachusetts, USA). All peptides were mixed at a concentration of 60 fmol/µL and added to each 1 µg of digested peptide sample before analysis by LC-MS/MS. 18 proteins were selected based on derivation cohort and literature. One or two peptides were selected for each protein (see online supplemental table 10). The specificity of the peptide selected for the protein was confirmed by Basic Local Alignment Search Tool (BLAST) search against the UniProtKB/Swiss-Prot database (https://blast.ncbi.nlm.nih.gov), which required that the peptide length should be more than four amino acids. For the whole proteome, analysis of the raw LC-MS/MS data was performed using MaxQuant (V.2.0.3.1) and the interrogation was carried out against the UniProtKB human database (V.2024–04). The validation of specific proteins was performed with Skyline.^6^ Briefly, for each peptide, three fragments ions were selected for heavy and light peptide identification and the most intense ion was selected with z=+2 or z=+3. Quantification was calculated using the weight-to-light ratio and normalised by the amount of heavy peptide added.

### Data visualisation, differential analysis, enrichment, statistical analysis and omics integration

All these analyses were performed with R and packages limma for differential protein expression analysis (V.3.52.4), DESeq2 for differential gene expression analysis (V.1.38.3) and mixOmics (V.6.20.0).^7^,^9^ Proteomics data were visualised by principal component analysis (PCA) and partial least squares-discriminant analysis (PLS-DA). All statistics tests on differential expression analyses were performed with a correction of p values with the Benjamini-Hochberg method (adjusted p<0.05).^10^ The whole transcriptomic and proteomic data sets were integrated using the DIABLO (Data Integration Analysis for Biomarker discovery using Latent cOmponent) R mixOmics framework. Sankey plot was created with SankeyMATIC on proteins identified in both cohorts and differentially expressed (adjusted p<0.05). Venn diagrams visualisations were created on Canva.

Functional enrichment and pathway analysis were conducted using Metascape (https://metascape.org/gp/index.html; V.3.5.20240901) with the Gene Ontology terms and Hallmark gene sets. Metascape uses the hypergeometric distribution (Fisher’s exact test) to identify all ontology terms that contain a statistically greater number of candidates in common with an input list than expected by chance. As recommended in Metascape guidelines, terms with a p value<0.01, a minimum of three candidates and an enrichment factor >1.5 were selected for visualisation.^11^

Boxplots and heatmap for AQUA peptides analysis were performed with GraphPad Prism (V.10.1.0, San Diego, California, USA). Box plots statistics were performed with the non-parametric Mann-Whitney test (p<0.05). For the heat map, min/max normalisation was used to represent the mean of the average peptide intensities for each protein.

Proteomics data sets analysed during the current study are available through Proteomic Xchange (data set number: PXD049364 (derivation cohort) and PXD057210 (validation cohort)) and transcriptomics data sets through SRA depository from NCBI:PRJNA1188072.

## Results

### Molecular signatures of EC exposed to IgG from patients with SSc in the derivation cohort

Patients’ characteristics are described in online supplemental table 1. EC were exposed to purified IgG, positive stimuli (IL-1β, TNF-α and LPS) or none (negative control). Cell lysates were analysed in LC-MS/MS and 3’ messenger RNA (mRNA) sequencing.

**Figure 1:**
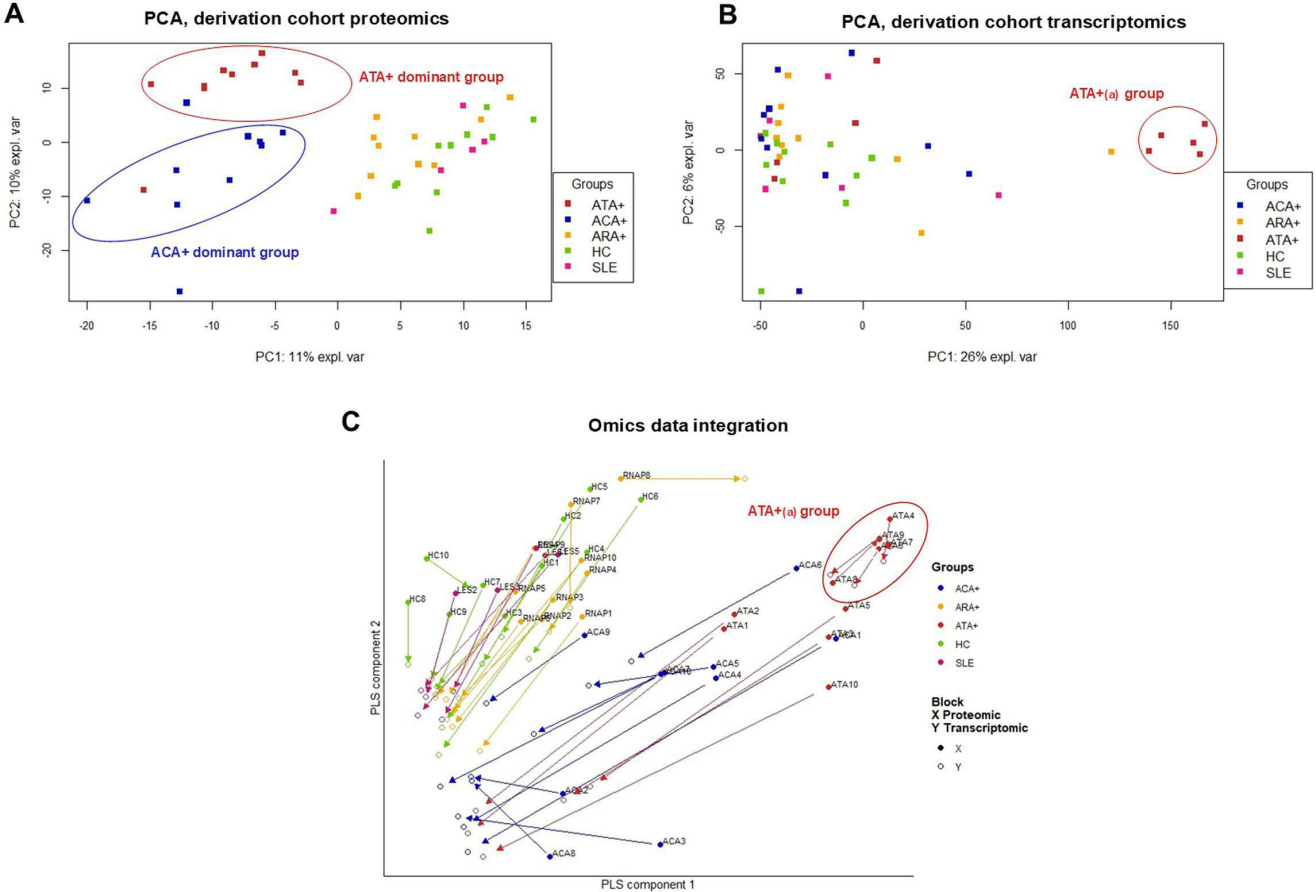
Effect of total purified IgG from derivation cohort on EC omics profiles. PCA scatter plots for the analysed cell samples. (**A**) PCA highlights EC proteins assessed by LC-MS/MS and (**B**) mRNA expressions assessed by 3’ mRNA sequencing according to ANA serotypes. (**C**) The arrow plot represents variation between proteomics and transcriptomics in each individual. ATA(a) group expressed less variation and appeared more homogeneous. ACA+, anti-centromere positive patients; ANA, antinuclear antibodies; ARA+, anti-RNA polymerase-III positive patients; ATA+, anti-topoisomerase-I positive patients; EC, endothelial cells; HC, healthy controls; LC-MS/MS, liquid chromatography with tandem mass spectrometry; mRNA, messenger RNA; PCA, principal component analysis; PLS, partial least squares; SLE, patients with systemic lupus erythematosus.

**Figure 2:**
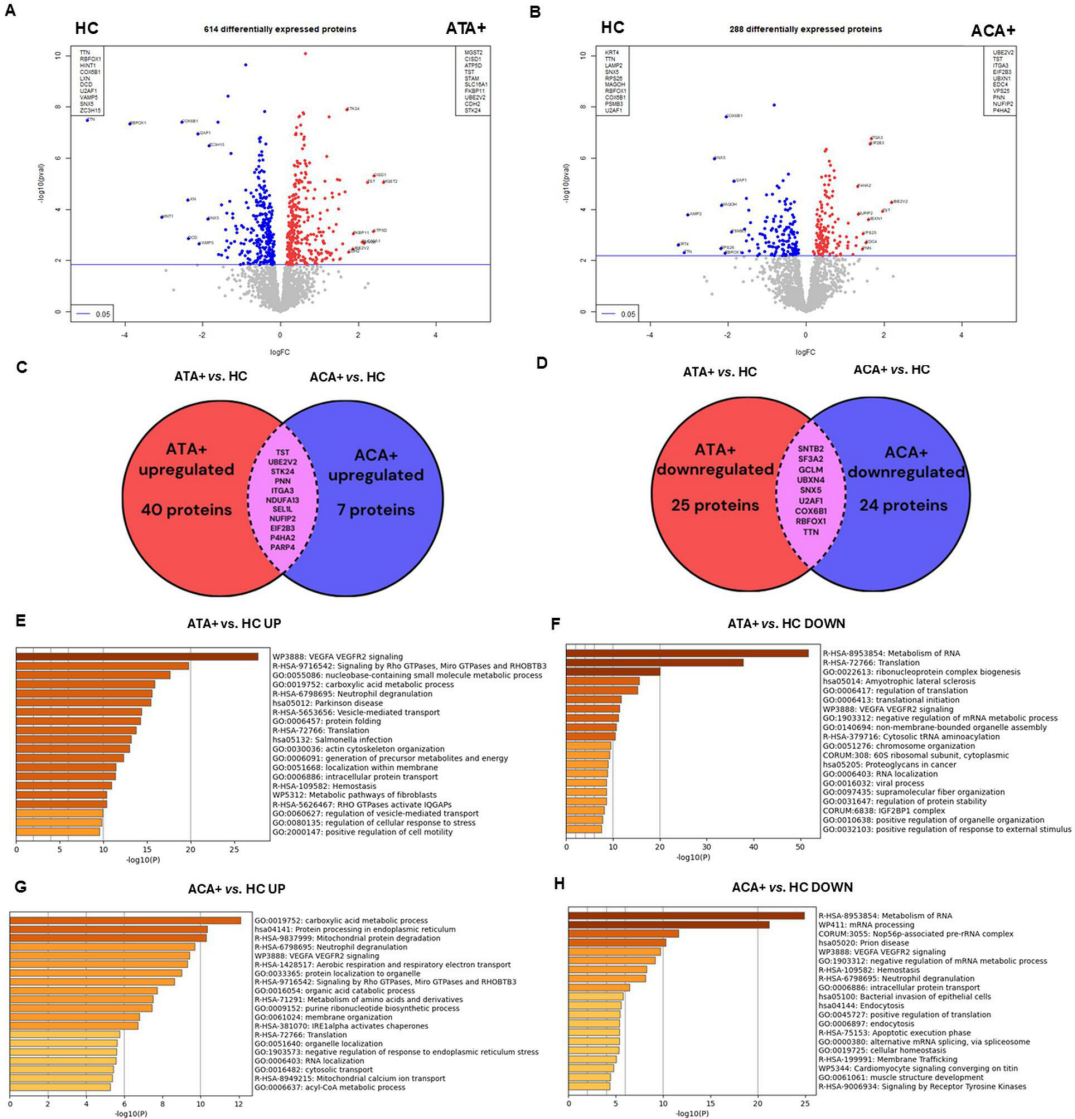
Effect of total purified IgG ATA+ and ACA+ on endothelial cells proteome in derivation cohort. Volcano-plots represent differential analysis (**A**) between ATA+ versus HC and (**B**) ACA+ versus HC comparisons. Commonly (**C**) overexpressed and (**D**) underexpressed proteins in the comparisons ATA+ versus HC and ACA+ versus HC are provided. Enrichment analysis in (**E**) upregulated DEP and (**F**) downregulated DEP in ATA+group. Enrichment analysis in (**G**) upregulated DEP and (**H**) downregulated DEP ACA+group. ACA+, anti-centromere positive patients; ATA+, anti-topoisomerase-I positive patients; DEP, differentially expressed proteins; HC, healthy controls.

Proteomics identified and quantified 2141 expressed proteins in all samples. First, we visualised the EC proteome under all conditions. Non-stimulated EC appeared largely distinct from EC in the presence of positive controls or IgG (online supplemental figure 1A), substantiating different molecular responses in EC. We then processed the data without controls to better explore the effect of IgG on EC. PCA showed three distinct groups of subjects: a first one including mostly ATA+patients, a second one including mostly ACA+patients and a third heterogeneous group comprising ARA+patients, patients with SLE and HC (figure 1A). The comparison of EC proteome in the presence of purified IgG from ATA+patients versus HC or from ACA+patients versus HC revealed 614 and 288 differentially expressed proteins (DEP), respectively (figure 2A,B and online supplemental table 2). 11 proteins such as integrin alpha-3 (ITGA3) were overexpressed while 9 proteins were underexpressed both in ATA+ and ACA+groups (figure 2C,D). A complete list of DEP is available in online supplemental table 3. In the ATA+group, overexpressed DEP were enriched in vascular endothelial growth factor A (VEGFA) and vascular endothelial growth factor receptor type 2 (VEGFR2) signalling and in signalling by Rho GTPases whereas underexpressed proteins were enriched in the metabolism of RNA and translation (figure 2E,F). In the ACA+group, overexpressed DEP were enriched in carboxylic acid metabolic process and in proteins processing in reticulum endoplasmic whereas underexpressed proteins were enriched in the metabolism of RNA and mRNA processing (figure 2G,H). No DEP were identified when comparing ARA+patients versus HC and patients with SLE versus HC (online supplemental table 2).

**Figure 3:**
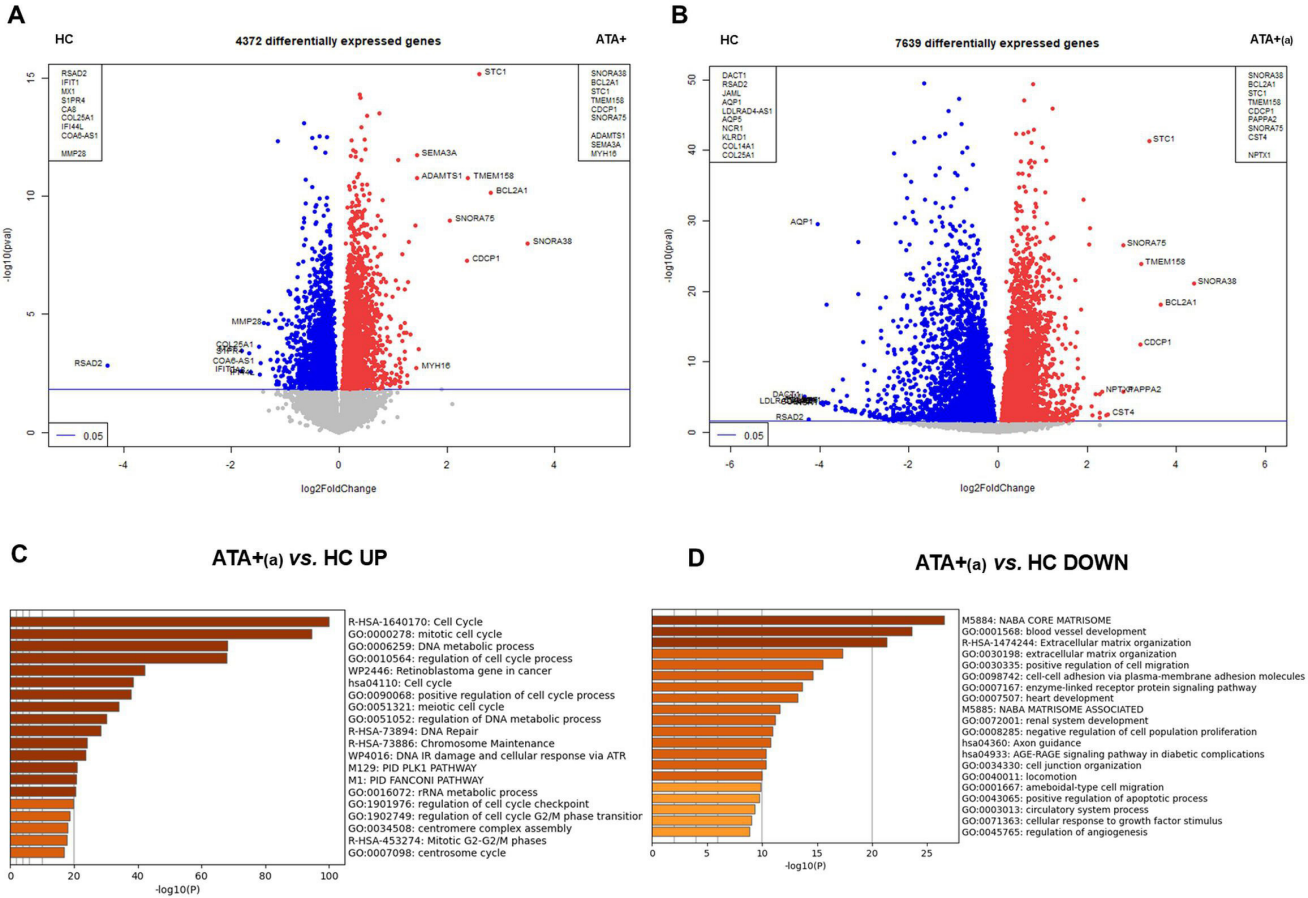
Effect of total purified IgG ATA+ on endothelial cells transcriptome in the derivation cohort. Volcano-plots represent differential analysis between (**A**) ATA+ versus HC (**B**) ATA+(a) versus HC. Enrichment analysis of (**C**) upregulated DEG in ATA+(a) versus HC and (**D**) downregulated DEG in ATA+(a) versus HC. ATA+, anti-topoisomerase-I positive patients; ATA+(a), anti-topoisomerase-I positive patients group a; DEG, differentially expressed genes; HC, healthy controls.

Transcriptomics identified 16 805 mRNA. Visualising all conditions, EC in the presence of positive controls (IL-1β, TNF-α and LPS) and EC in the presence of IgG or non-stimulated EC appeared largely distinct (online supplemental figure 1B). We then analysed the data without controls to better explore the effect of IgG on EC transcriptome. PCA distinguished a specific group of five ATA+patients (ATA+_(a)_ group) clearly separated from five other ATA+patients (ATA+_(b)_ group) mixed with ACA+patients, ARA+patients, patients with SLE and HC (figure 1B). We performed differential analysis on transcriptomics data (online supplemental table 4) with a specific focus on the ATA+_(a)_ and ATA+_(b)_ groups. The comparison of EC transcriptome in the presence of purified IgG from ATA+ versus HC, ATA+_(a)_ versus HC, ATA+_(b)_ versus HC, ATA+_(a)_ versus ATA+_(b)_ groups revealed 4372, 7639, 145 and 6839 differentially expressed genes (DEG), respectively (figure 3A,B, online supplemental table 4). The ATA+_(a)_ group contributed the most to the effect of IgG from ATA+patients on the EC transcriptome. In ATA+_(a)_ versus HC and ATA+_(a)_ versus ATA+_(b)_, 3014 DEG were commonly upregulated; among them BCL2-related protein A1 (BCL2A1) and stanniocalcin-1. 2775 DEG were commonly downregulated; among them toll-like receptor 4 (TLR4) and aquaporin 1. A detailed list of DEG is available in online supplemental table 5. In ATA+_(a)_ group, upregulated DEG were enriched in cell and mitotic cell cycle and in DNA metabolic process and downregulated DEG were enriched in blood vessel development and extracellular matrix organisation (figure 3C,D).

To allow a comprehensive representation and analysis of these findings, we performed omics data integration combining proteomic and transcriptomic profiles. 2041 variables were common to both data sets. The arrow plot representing data integration distinguished three groups of subjects: a first stable group composed by ATA+_(a)_ patients with low variability between proteomic and transcriptomic profiles, a second group composed by ATA+_(b)_ and ACA+patients with higher variability and a third group composed by ARA+patients, patients with SLE and HC (figure 1C). These findings reinforced the existence of two ATA+groups: one stable, homogeneous (ATA+_(a)_ group) distinct from the other one more heterogeneous and mixed with the ACA+group (ATA+_(b)_ group). Intrigued by these results, we sought to confirm the profiles of patients with ATA in another SSc cohort.

### Proteomic profiles of EC in the presence of IgG from patients with SSc in the validation cohort

Patients’ characteristics are described in online supplemental table 6. EC were exposed to purified IgG and cell lysates were analysed in LC-MS/MS.

**Figure 4:**
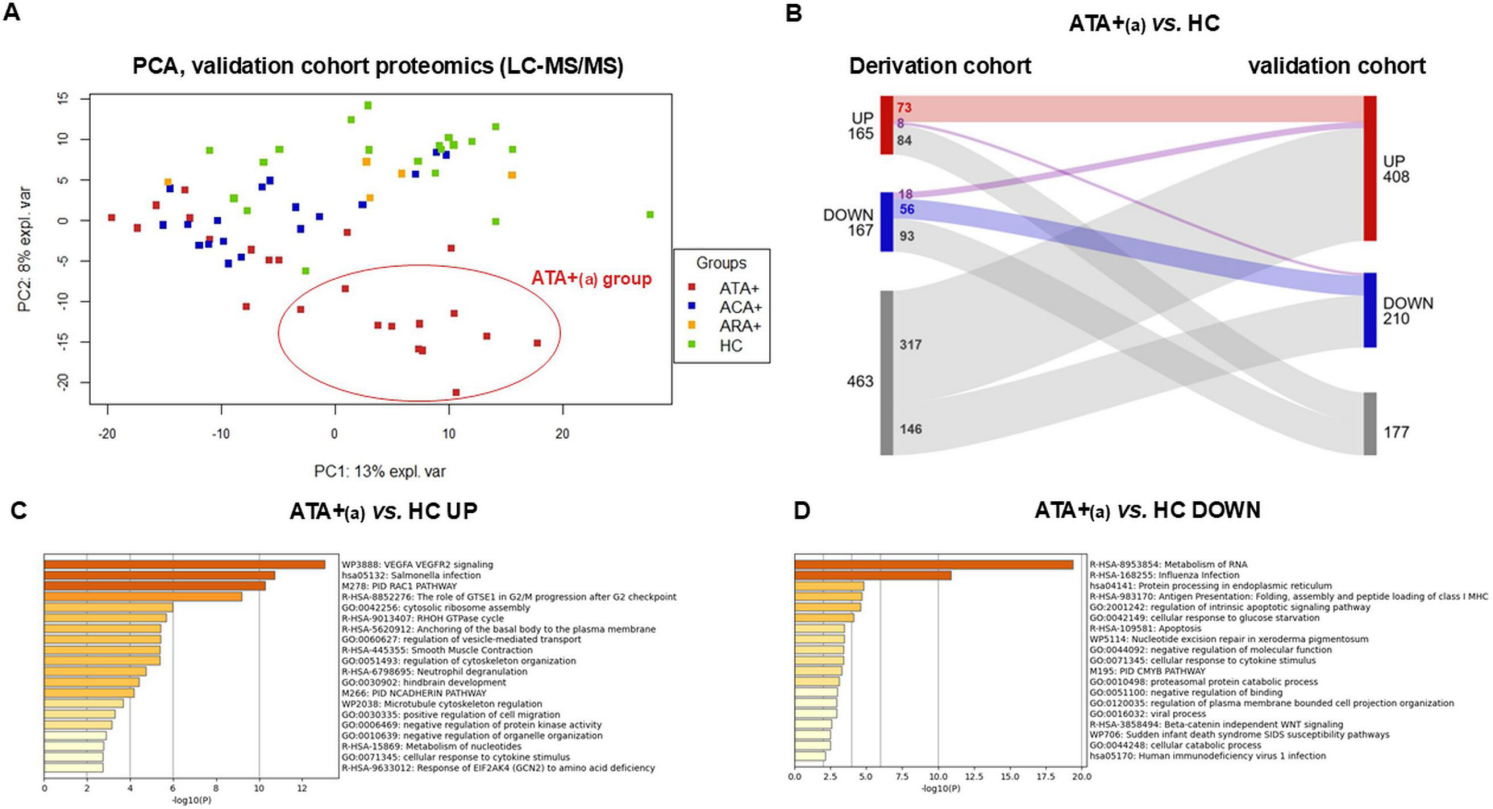
Effect of total purified IgG from on EC proteome in the validation cohort. PCA scatter plots for the analysed cell samples. (**A**) PCA highlights EC proteins expression assessed by LC-MS/MS according to ANA serotypes in the validation cohort. (**B**) Sankey plot represents commonly overexpressed and underexpressed proteins among derivation and validation cohort. Enrichment analysis of (**C**) commonly upregulated DEG in ATA+(a) versus HC and (**D**) commonly downregulated DEG in ATA+(a) versus HC in both cohorts. ACA+, anti-centromere positive patients; ARA+, anti-RNA polymerase-III positive patients; ATA+, anti-topoisomerase-I positive patients; ATA+_(a)_, anti-topoisomerase-I positive patients group a; EC, endothelial cells; HC, healthy controls; LC-MS/MS, liquid chromatography with tandem mass spectrometry; PCA, principal component analysis.

Proteomics identified and quantified 2153 proteins. Among them, 1666 (77 %) had already been identified and quantified in the derivation cohort. PCA showed three distinct groups of subjects: one group composed only of 11 ATA+patients (ATA+_(a)_ group) and a second heterogeneous group composed of others ATA+patients (ATA+_(b)_ group), ACA+patients and HC (figure 4A).

We performed a similar differential analysis than those in the derivation cohort (onlinesupplemental tables 7  8). The comparison of EC proteome in the presence of purified IgG from ATA+ versus HC, ATA+_(a)_ versus HC, ATA+_(b)_ versus HC, ATA+_(a)_ versus ATA+_(b)_ and ACA+ versus HC revealed 403, 732, 255, 903 and 149 DEP, respectively (online supplemental figure 2A,B). Venn diagram displaying up and down DEP confirmed that the ATA+_(a)_ group was the most distinct (online supplemental figure 2C,D). In the ATA+_(a)_ versus HC comparison, overexpressed proteins were enriched in intracellular protein transport, metabolism of RNA and VEGFA VEGFR2 signalling and underexpressed proteins were enriched in mRNA processing (online supplemental figure 2 E,F). In the ACA+ versus HC comparison, overexpressed proteins were enriched in actin filament-based process and mRNA processing and underexpressed proteins were enriched in protein processing in endoplasmic reticulum (online supplemental figure 2 G,H).

We explored the similarity between ATA+_(a)_ groups in derivation and validation cohorts using a Sankey plot (figure 4B). 73 proteins were commonly overexpressed in ATA+_(a)_ versus HC in both cohorts, among them VE-cadherin (CDH5). These proteins were enriched in VEGFA VEGFR2 signalling (figure 4C). 56 proteins were commonly underexpressed in ATA+_(a)_ versus HC in both cohorts (figure 4B) and were enriched in metabolism of RNA and protein processing in reticulum endoplasmic (figure 4D). Of note, only 8 proteins were overexpressed in ATA+_(a)_ versus HC in the derivation cohort while underexpressed in the validation cohort, and 18 were underexpressed in the derivation cohort while overexpressed in the validation cohort (figure 4B).

### FA contribution in the observed omics profiles

**Figure 5:**
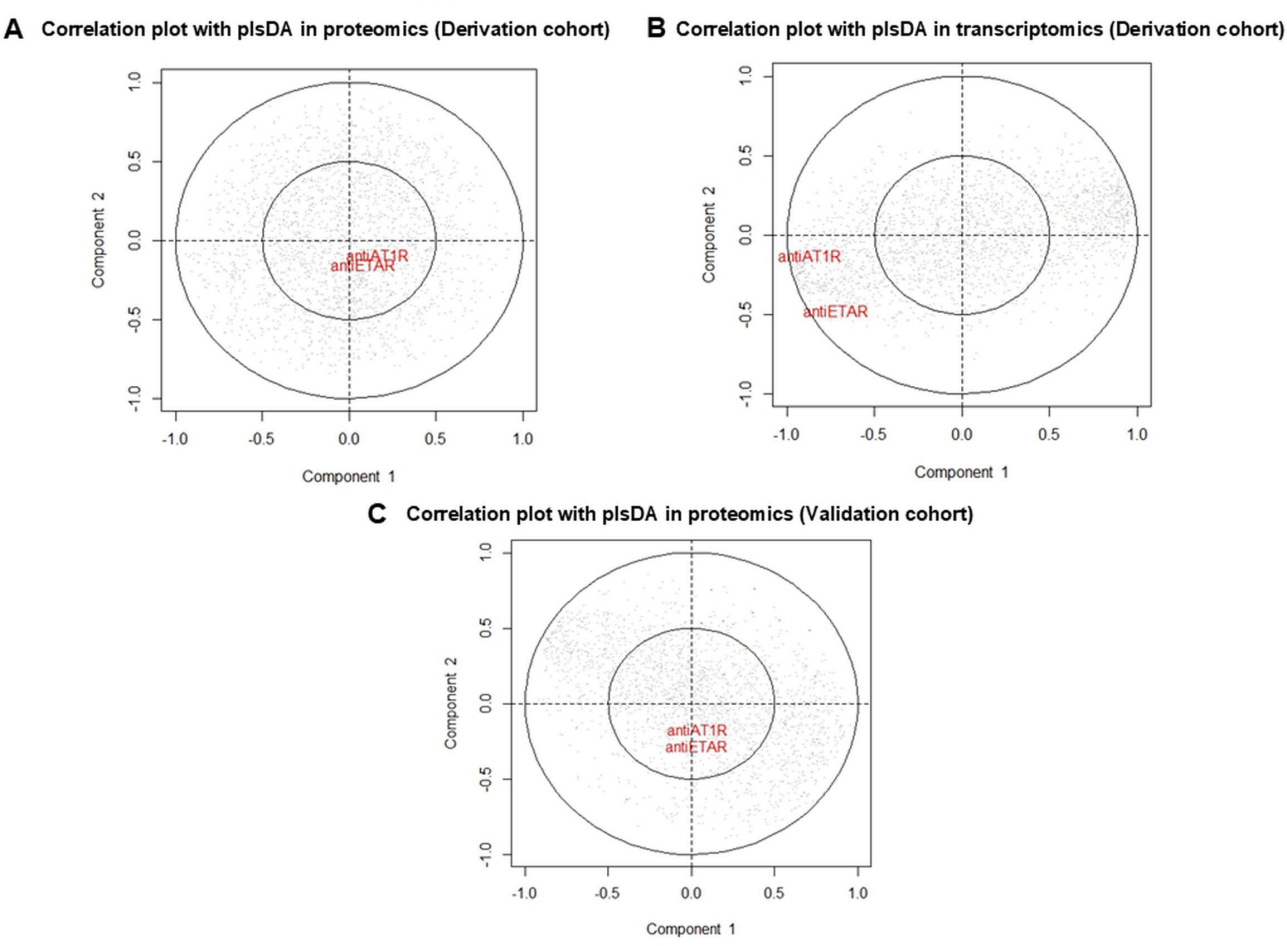
Impact of FA on omics profiles. PLS-DA axes according to FA levels in (**A**) derivation cohort proteomic profiles, (**B**) derivation cohort transcriptomic profiles and in (**C**) validation cohort proteomic profiles. AT1R, angiotensin II type 1 receptor autoantibodies; ETAR, endothelin-1 type A receptor autoantibodies; FA, functional antibodies; PLS-DA, partial least squares-discriminant analysis.

We examined the influence of FA in the obtained omics profiles. In the derivation cohort, AT1R and ETAR Aab were present in patients with SSc among the different serotypes (online supplemental figure 3A,B). FA levels were not significantly correlated with PLS-DA axes (figure 5A), suggesting that FA did not impact proteomic signatures. The observed correlation with the PLS-DA axes of the transcriptomics data indicated that FA influenced the EC transcriptome, among many other contributing factors (figure 5B). In the validation cohort, AT1R and ETAR Aab were not different between patients with SSc and HC. FA levels were not significantly correlated with PLS-DA axes (figure 5C and online supplemental figure 3C).

### ATA+_(a)_ and ATA+_(b)_ group characteristics

To better characterise the ATA+groups, we compared clinical data in ATA+_(a)_ and ATA+_(b)_ from the validation cohort. ILD was more frequent in ATA+_(a)_ group (100% vs 54%, p value: 0.008). Moreover, ATA+_(a)_ group was characterised by the association of ILD and digital ulceration (DU) (p value: 0.014) (table 1).

**Table 1:** ATA+groups comparisons (validation cohort)

	ATA+(a) group (N=11)	ATA+(b) group (N=13)	P value
Age at inclusion, mean (SD) years	59.4 (13.2)	62.7 (13.1)	0.564
Disease duration at inclusion, mean (SD) years	6.1 (4.9)	6.4 (5.7)	0.424
mRSS at inclusion, mean (SD)	6.1 (3.2)	7.4 (4.9)	0.585
DU, n (%)	7 (63.6)	4 (30.8)	0.117
ILD, n (%)	11 (100)	7 (53.8)	**0.008**
ILD or DU, n (%)	11 (100)	9 (62.2)	**0.045**
ILD+DU, n (%)	7 (63.6)	2 (15.4)	**0.014**

Quantitative variables are represented as mean and standard deviation (SD. Bold value represent significant P value

ATA+, anti-topoisomerase-I positive patients; ATA+(a), ATA+group a; ATA+(b), ATA+group b; mRSS, modified Rodnan skin score; ILD, interstitial lung disease; DU, digital ulceration.

### Quantification of selected proteins in the validation cohort

**Figure 6:**
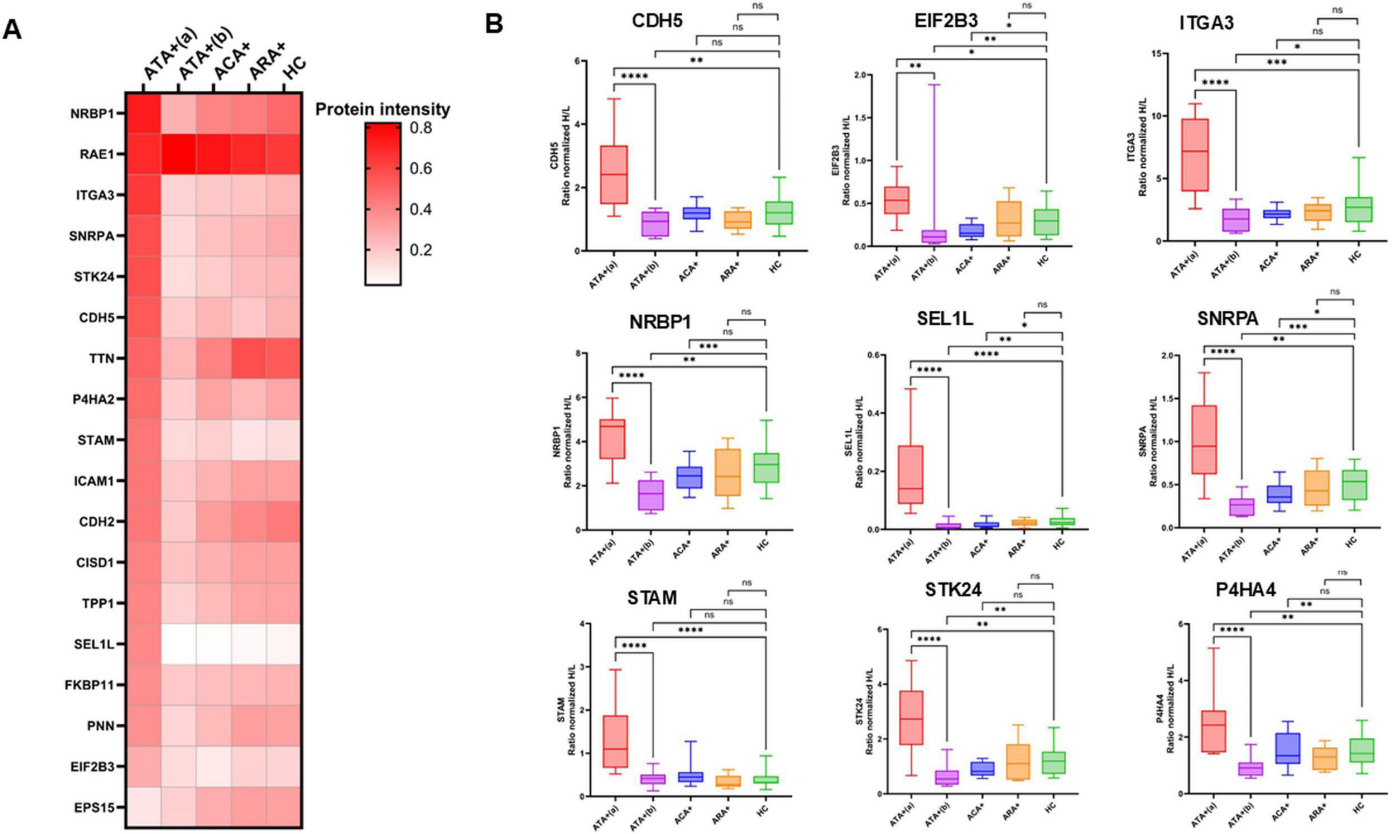
Targeted proteomics by automated quantification proteomics approach further characterised ATA+(a) and ATA+(b) group (validation cohort). (**A**) Heatmap represents selected proteins expression among groups. (**B**) Boxplots represent detailed overexpressed proteins in ATA+(a) group both vs ATA+(b) and HC (**B**). NS: non-significant (p value>0.05); *p value<0.05; **p value<0.01; ***p value<0.001; ***p value<0.0001. ATA+, anti-topoisomerase-I positive patients (N=24); ATA+(a), ATA+group a (N=11); ATA+(b), ATA+group b (N=13); ACA+, anti-centromere positive patients (N=18); ARA+, anti-RNA polymerase-III positive patients (N=5); ATA+, anti-topoisomerase-I positive patients; ATA+_(a)_, anti-topoisomerase-I positive patients group a; ATA+_(b)_, anti-topoisomerase-I positive patients group b; CDH2, cadherin 2; CDH5, VE-cadherin; CISD1, CDGSH iron sulphur domain 1; EIF2B3, eukaryotic translation initiation factor 2B subunit gamma; EPS15, epidermal growth factor receptor pathway substrate 15; FKBP11, FKBP prolyl isomerase 11; HC, healthy controls (N=20); ICAM-1, intercellular adhesion molecule 1; ITGA3, integrin alpha-3; NRBP1, nuclear receptor-binding protein; P4HA2, prolyl 4-hydroxylase subunit alpha-2; PNN, desmosome associated protein; RAE1, Rae1 RNA export 1; SEL1L, adaptor subunit of SYVN1 ubiquitin ligase; SNRPA, small nuclear ribonucleoprotein polypeptide A; STAM, signal transducing adaptor molecule; STK24, serine/threonine-protein kinase 24; TPP1, tripeptidyl-peptidase 1; TTN, titin.

Finally, we derived a set of 18 representative proteins and quantified them in the validation cohort using mass spectrometry-based AQUA technology (panel of confirmed proteins are available in online supplemental table 9) and detailed proteins quantification are available in online supplemental table 10. It confirmed that ATA+_(a)_ was distinct from ATA+_(b)_, ACA+, ARA+ or HC (figure 6A). PCA based on quantified proteins confirmed ATA+_(a)_ as the most distinct and homogeneous group of subjects (online supplemental figure 4A). 13 proteins were significatively overexpressed in ATA+_(a)_ versus ATA+_(b)_ or HC and 9 overexpressed in both comparisons ATA+_(a)_ versu ATA+_(b)_ and HC including serine/threonine-protein kinase 24 (STK24), ITGA3, CDH5, nuclear receptor-binding protein, adaptor subunit of SYVN1 ubiquitin ligase (SEL1L) and eukaryotic translation factor 2B subunit alpha subunit gamma (EIF2B3) (figure 6B). Among them, STK24, ITGA3 and SEL1L appeared associated to dcSSc, ILD and DU (online supplemental figure 4B).

## Discussion

This study revealed that purified IgG from patients with SSc can modify the EC proteome and transcriptome according to ANA serotypes. FA were present in patients with SSc but seemed to have a minimal impact on omics profiles. In the derivation cohort, IgG from ATA+patients induced a distinct EC proteomic profile. In transcriptomics, the ATA+group was further divided into two groups of subjects. The validation cohort confirmed a singular proteomic profile induced by IgG from ATA+patients on EC, with the existence of two ATA+groups, including one with a more pronounced effect on EC, where organ involvement such as ILD and DU were more often present. Quantitative proteomics supported these findings providing most upregulated or downregulated proteins in EC exposed to purified IgG from ATA+patients.

ANA are present in the majority of SSc cases and are a key tool in disease management. Three of them (ATA, ACA and ARA) are part of the last ACR/ELUAR classification criteria.^4^ In fact, these are generally exclusive to the same patient and are associated with different SSc clinical profiles (lcSSc or dcSSc, internal organs involvement) and severity.^1 2^ Moreover, ANA explained part of the groups of patients in several cluster analysis studies, reinforcing their crucial role in catching SSc complexity.^12 13^ Nevertheless, the role of ANA in SSc pathophysiology remains partially unknown. Several observational data of ANA pathogenicity in SSc emerged. ANA precede for years SSc onset^14 15^ and the presence of ATA and ACA in patients with undifferentiated connective tissue disease points to the evolution of definite SSc.^16^ Moreover, ANA title correlate with disease severity and progression^17^,^19^ and molecular profiles are associated with ANA serotypes.^20 21^ Finally, patients with SSc may respond response to therapeutic targeting humoral immunity such as rituximab,^22 23^ and more recently anti-CD19 chimeric antigen receptors T cells.^24 25^

In the present study, EC omics profiles differed according to ANA serotypes in both cohorts. IgG from ATA+patients induced the most singular proteomic profile in EC characterised by VEGFA and VEGFR2 signalling enrichment in up and downregulated proteins, and VE-cadherin was overexpressed in EC in the presence of IgG ATA+ in both cohorts. IgG from ACA+patients induced DEP enriched in VEGFA and VEGFR2 signalling in derivation cohort but was not retrieved in validation cohort. SSc vasculopathy is characterised by disturbed vessel morphology with enlarged capillaries and an overall reduction in capillary density with respect to the expression of both VEGF and its receptors, VEGFR-1 and VEGFR-2.^26^ VE-cadherin, an endothelium junction protein, plays an important role in vascular development. During angiogenesis, VEGFR2 binds to VE-cadherin and VEGFR2 kinase activity leads to VE-cadherin phosphorylation, which induces cell survival and proliferation through the PI3K/AKT signalling pathway.^27^ Interestingly, Raschi *et al*, showed that immune complexes from patients with SSc containing ATA upregulated TLR4 expression and increased activation of SAPK-JNK, p38MAPK and Akt signalling via an Fc-gamma-receptor independent mechanism which engaged endothelial damage.^28^ This data suggests angiogenesis promotion by IgG from ATA+patients on EC and needs to be further confirmed. In SSc, EC are known to undergo a mesenchymal transition reducing angiogenesis in which VE-cadherin expression decreased.^29^

We highlighted two groups of ATA+patients according to the modification induced on EC transcriptome and proteome. The most homogeneous ATA+group (ATA+_(a)_) strongly modified EC omics profiles and was characterised by a higher frequency of severe organ involvement (especially ILD). In a large cluster analysis of 6927 patients with SSc, we characterised six groups of patients with SSc based on clinical features, ANA profile and mortality. ATA+patients were the majority in three of these groups, with different phenotypic and evolutionary profiles. The group with the highest proportion of ATA+patients had the most severe disease course.^12^ In the present study, heterogeneity among ATA+patients was explained by distinct EC omics profiles. Interestingly, a recent study based on transcriptomics, metabolomics and flow cytometry in whole blood demonstrated that patients with SSc exhibit distinct molecular signatures according to their ANA serological status.^30^ This may suggest, considering the present study, a contribution of IgG to the observed molecular signatures.

The EC transcriptome in the presence of IgG from ATA+_(a)_ group was characterised by B-cell lymphoma 2A2 overexpression and was enriched in mitotic cell cycle and in DNA metabolic process. BCL2A1, a member of the BCL2 family, is overexpressed in various cancer cells and may contribute to tumour progression.^31^ In EC, BCL2 family possesses an anti-apoptotic effect, dependent and independent of VEGF.^32 33^

In the validation cohort, the targeted proteomics approach using AQUA technology allowed precise quantification of proteins expression.^34^ This confirmed the result of exploratory analyses in derivation and validation cohorts by clearly separating ATA+_(a)_ and ATA+_(b)_ groups. IgG from ATA+groups might promote the opposite effect. For example, IgG from ATA+_(a)_ group induced overexpression of VE-cadherin which is hallmarks of angiogenesis, whereas IgG from ATA+_(b)_ group decreased VE-cadherin expression.^35^ The precise meaning of these omics profiles need to be further explored. Interestingly, IgG from ATA+_(a)_ patients were able to induce overexpression of proteins known to be the target of newly identified SSc Aab. Indeed, IgG from ATA+_(a)_ group induces overexpression of EIF2B3 and SNRPA. Anti-EIF2B Aab are found in 1–2% of patients with SSc and are associated with dcSSc, arthralgia and ILD.^36 37^ Anti-SNRPA are newly described, might be present in 11% of patients with SSc, are more often present in ATA-negative patients and are associated with PAH.^38^ Finally, ITGA3, SEL1 and STK24 were overexpressed in ATA+_(a)_ and correlated with dcSSc, DU and ILD. Integrins family genes expression in SSc skin correlates with modified Rodman skin score and might promote skin fibrosis by affecting extracellular matrix turnover.^39^ STK24 was recently known to promote tumorigenesis in lung cancer through STAT3/VEGFA signalling pathway.^40^

FA are described in SSc and might have a pathogenic role especially in vasculopathy.^41^ AT1R and ETAR Aab are found in SSc and are associated with disease-related complications and mortality. Both might be pathogenic by binding to their respective receptor-inducing signal-regulated kinase 1/2 phosphorylation and increasing transforming growth factor β gene expression in EC.^42^ Immunised mice by membrane-embedded human AT1R developed perivascular skin and lung inflammation, lymphocytic alveolitis, weak lung endothelial apoptosis and skin fibrosis. Moreover, passive transfer into mice with monoclonal AT1R antibody provoked skin and lung inflammation, which was not observed in angiotensin receptor a/b receptor knockout mice, via monocyte activation leading to the production of profibrotic markers by dermal fibroblasts.^43^ FA were present in both SSc cohorts but were also present in some HC and patients with SLE. In the present study, AT1R and ETAR Aab did not explain the EC proteomic profiles (both in the derivation and validation cohorts) induced by SSc IgG and could contribute to EC transcriptomic profiles. Bankamp *et al* found by using luminometric assay that 52% of patients with SSc had functional active AT1R Aab (stimulatory or inhibitory capacity) but AT1R Aab were also present in others autoimmune diseases and HC and did not correlate with organ involvement. Functional AT1R Aab detected by luminometric did not correlate with AT1R and ETAR Aab levels in ELISA assay whereas there was a strong correlation between AT1R Aab, ETAR Aab and ATA levels.^44^

In our study, EC changes induced by IgG from patients with SSc were mostly independent of the presence of FA suggesting a contribution of ANA particularly ATA or immune complexes containing ATA. IgG from ATA+patients inhibited TOPO-I in vitro, and we previously showed that IgG ATA+induced profibrotic profiles in normal dermal fibroblasts.^5 45^ Pathogenic mechanisms of ANA in SSc such as interaction between immune complexes or Aab are suspected.^46 47^ Nevertheless, the capacity of ANA to directly interact with their intracellular antigens remains largely unknown.^2^ In SLE, anti-DNA has been reported to penetrate cells and interact with intracellular antigen.^48^ Recently, it was shown that anti-Mi2 and anti-PM/Scl Aab accumulated in the nucleus of myofibers from patients with myositis and that their internalisation into healthy myoblasts induced transcriptomic profiles similar to those observed in the muscles of patients with the same ANA serotype. The modification of the myoblast profiles was consecutive to the autoantibody-autoantigen interaction.^49^ Finally, May *et al* recently showed that a monoclonal ATA produced by a single B cell from patient with SSc can penetrate living colonic cancer cells, localised into nuclei and then inhibits formation of the TOPO-I cleavage complex necessary for DNA nicking.^50^

The sample size of the derivation cohort precluded the study of clinical associations between groups. The use of total purified IgG rather than specific purified Aab prevented any firm conclusion regarding ANA pathogenicity but should be seen as an essential step before future assessments requiring a high volume of antibodies. The strengths of this study include multiomics analysis in the derivation cohort and the use of omics integration data, which reinforced exploratory capacity. Proteomic was confirmed in a validation cohort, and a sensitive method was used to quantify discriminant targets belonging to the derivation cohort molecular signatures.

## Conclusion

IgG from patients with SSc modified the EC proteome and transcriptome profiles in an ANA serotype-dependent manner. FA was present in patients with SSc but no association between the presence of FA and change in proteome was observed whereas IgG from ATA+patients induced singular and distinct EC profiles. A subgroup of ATA+patients, whose IgG strongly modified EC proteome and transcriptome, was characterised by more frequent severe organ involvement. The potential role of ANA in SSc needs to be confirmed in further studies.

## Supplementary material

10.1136/rmdopen-2024-004290 online supplemental file 1

10.1136/rmdopen-2024-004290 online supplemental file 2

10.1136/rmdopen-2024-004290 online supplemental file 3

10.1136/rmdopen-2024-004290 online supplemental file 4

10.1136/rmdopen-2024-004290 online supplemental file 5

## Data Availability

Data are available in a public, open access repository. Data are available upon reasonable request.
