# Supplementary material for: Immunoglobulins G from Patients with Systemic Sclerosis Modify the Molecular Signatures of Endothelial Cells
Source: RMD Open. 2025 Mar 23;11(1):e004290. doi: 10.1136/rmdopen-2024-004290 (PMC11931898; doi:10.1136/rmdopen-2024-004290)
Supplement: online supplemental file 1 [file rmdopen-11-1-s001.pdf]

Group	Sex	Age at inclusion	Disease duration at inclusion (years)	ENA type	Cutaneous subtype	IgG level (mg/mL)	mRSS at study inclusion	Organs Involvement	Use of immunosuppressive therapy	ATA+ group (transcriptomics)
**ATA+**	F	74	13	ATA	dcSSc	10.99	18	ILD<br>DU<br>Telangiectasias	EDX<br>MTX	ATA+(b)
	M	52	3	ATA	dcSSc	10.39	11	ILD<br>telangiectasias	MMF<br>CYC<br>TCZ	ATA+(b)
	M	42	15	ATA	dcSSc	9.10	28	ILD<br>DU<br>telangiectasis	MTX<br>MMF<br>CYC<br>MPA<br>RTX	ATA+(b)
	F	29	7	ATA	dcSSc	8.49	7	ILD	CYC<br>RTX<br>MMF	ATA+(a)
	F	30	3	ATA	dcSSc	13.10	9	ILD<br>telangiectasias	MMF	ATA+(b)
	F	54	24	ATA	dcSSc	8.48	12	ILD<br>telangiectasias	None	ATA+(a)
	F	31	5	ATA	dcSSc	10.77	8	ILD<br>DU<br>Renal Crisis<br>telangiectasias	MMF<br>MPA	ATA+(a)
	M	77	10	ATA	dcSSc	8.86	5	ILD	MMF	ATA+(a)
	F	44	22	ATA	dcSSc	6.43	6	ILD<br>DU<br>telangiectasias	None	ATA+(a)

	M	55	10	ATA	dcSSc	8.96	12	ILD<br>PAH<br>telangiectasias	MMF	ATA+(b)
**ACA+**	F	82	2	ACA	lcSSc	9.69	4	PAH	None	
	F	71	8	ACA	lcSSc	10.28	2	PAH<br>telangiectasias	None	
	F	52	3	ACA	lcSSc	9.73	6	PAH<br>telangiectasias	None	
	F	72	21	ACA	lcSSc	6,93	18	PAH<br>telangiectasias	None	
	F	71	8	ACA	lcSSc	11.02	10	PAH<br>telangiectasias	None	
	F	38	9	ACA	lcSSc	7.62	1	telangiectasias	None	
	F	68	2	ACA	lcSSc	5.22	8	PAH<br>telangiectasias	None	
	F	52	5	ACA	lcSSc	11.15	4	None<br>telangiectasias	None	
	F	79	7	ACA	lcSSc	11.37	0	None<br>telangiectasias	None	
	F	37	8	ACA	lcSSc	11.41	2	DU<br>telangiectasias	None	
**ARA+**	F	50	8	ARA	dcSSc	3.79	5	ILD<br>telangiectasias	MMF<br>MPA	
	F	59	11	ARA	dcSSc	9.96	16	None<br>telangiectasias	MMF	
	F	75	3	ARA	dcSSc	11.72	9	None<br>telangiectasias	MMF	
	F	46	8	ARA	dcSSc	5.72	17	ILD<br>telangiectasias	MMF	
	F	58	23	ARA	lcSSc	4.84	0	telangiectasias	None	

	F	56	8	ARA	lcSSc	14.36	9	ILD<br>telangiectasias	None	
	F	43	7	ARA	dcSSc	10.32	13	ILD<br>telangiectasias	MMF	
	F	68	4	ARA	lcSSc	6.79	2	ILD<br>telangiectasias	MMF	
	F	54	10	ARA	lcSSc	13.69	6	DU	None	
	F	40	9	ARA	dcSSc	8.28	14	telangiectasias	MTX<br>MMF	
**HC**	F	61				5.44				
	F	61				9.03				
	F	60				7.87				
	F	60				8.26				
	H	59				14.15				
	F	63				8.01				
	F	63				6.44				
	F	55				10.20				
	F	55				11.00				
	F	49				9.54				
**SLE**	F	38	16	Anti-<br>DNA		9.81		Autoimmune<br>cytopenia<br>Myocarditis<br>LN<br>Cutaneous<br>Arthritis	MMF	
	F	24	3	Anti-<br>DNA		10.73		LN<br>Arthritis	MMF	
	F	39	22	Anti-<br>DNA		9.10		LN<br>Arthritis<br>Cutaneous	MMF<br>MTX	
	F	18	1	Anti-<br>DNA		8.19		LN<br>Arthritis	CYC<br>BEL	

								Cutaneous	MMF	
	F	20	5	Anti-DNA		13.16		Arthritis	MTX	

Supplemental Table 1: subjects characteristics (derivation cohort).

DcSSc: diffuse cutaneous systemic sclerosis; lcSSc: limited cutaneous systemic sclerosis; ATA+: anti-topoisomerase-I positive patients; ATA+(a): ATA+ group a; ATA+(b): ATA+ group b ; ACA+ anti-centromere positive patients; ARA+: anti-RNA polymerase-III positive patients; SLE: systemic lupus erythematosus patients; HC: healthy controls; ENA: extractable nuclear antigen; mRSS: modified Rodnan skin score; ILD: interstitial lung disease; PAH: pulmonary arterial hypertension; DU: digital ulceration; LN: lupus nephritis BEL: belimumab; CYC: cyclophosphamide; MMF: mycophenolate mofetil; MTX: methotrexate; MPA: mycophenolic acid; RTX: rituximab; TCZ: tocilizumab.

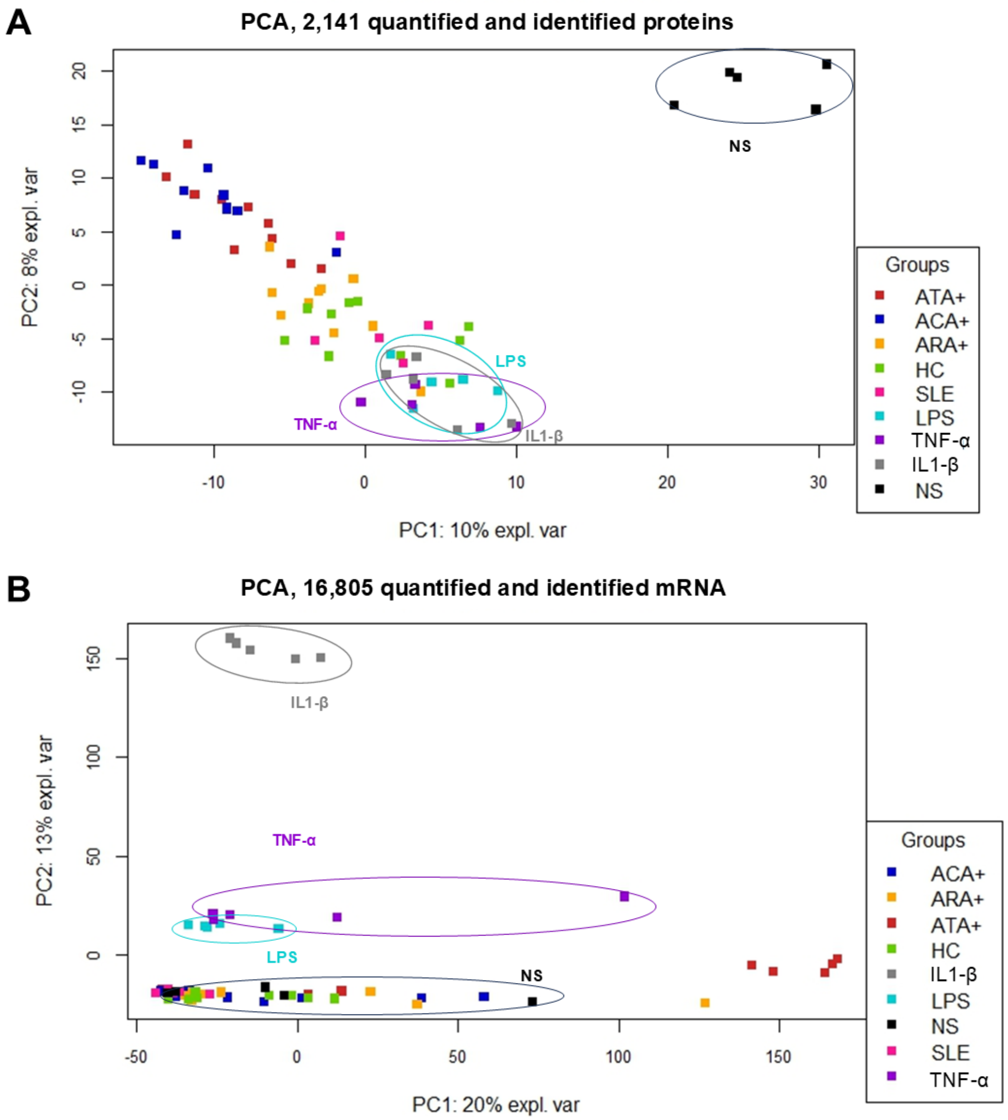
A
PCA, 2,141 quantified and identified proteins
PC2: 8% expl. var
PC1: 10% expl. var
NS
LPS
TNF-α
IL1-β
Groups
ATA+
ACA+
ARA+
HC
SLE
LPS
TNF-α
IL1-β
NS
B
PCA, 16,805 quantified and identified mRNA
PC2: 13% expl. var
PC1: 20% expl. var
IL1-β
TNF-α
LPS
NS
Groups
ACA+
ARA+
ATA+
HC
IL1-β
LPS
NS
SLE
TNF-α

Supplemental Figure 1: EC omics profiles in derivation cohort

PCA scatter plots for the analyzed cell samples. (A) PCA represents EC proteins assessed by LC-MS/MS and (B) mRNA expressions assessed by 3' mRNA sequencing in the presence of IgG and controls.

EC: endothelial cells; ATA+: anti-topoisomerase-I positive patients; ACA+: anti-centromere positive patients; ARA+: anti-RNA polymerase-III positive patients; SLE: systemic lupus erythematosus patients; HC: healthy controls; NS: non stimulated condition; IL1-β: interleukin 1 beta; TNF-α: tumor necrosis factor alpha; PCA: principal component analysis; LC-MS/MS: liquid chromatography with tandem mass spectrometry.

Comparison	**Total of DEP**	**UP (adj P Val<0.05)**	**UP (adj P Val<0.05), logFC>1**	**Down (adj P Val<0.05)**	**Down (adj P Val<0.05), logFC<-1**
ATA+ *vs.* HC	614	329	51	284	34
ATA+(b) *vs.* HC	314	170	35	143	28
ATA+(a) *vs.* HC	388	188	26	199	27
ATA+(a) *vs.* ATA+(b)	0	0	0	0	0
ACA+ *vs.* HC	288	147	18	141	33
ARA+ *vs.* HC	2	1	0	1	0
SLE *vs.* HC	0	0	0	0	0

Supplemental Table 2: Numbers of differentially expressed proteins in proteomics (derivation cohort).

DEP: differentially expressed proteins; UP: overexpressed DEP; DOWN: underexpressed DEP; LogFC: log Fold Change; ATA+: anti-topoisomerase-I positive patients; ACA+: anti-centromere positive patients; ARA+: anti-RNA polymerase-III positive patients; SLE: systemic lupus erythematosus patients; HC: healthy controls.

Supplemental Table 3: List of differentially expressed proteins in proteomics (derivation cohort).

Please refer to the Excel table.

Comparison	Total of DEG	UP (adj P Val<0.05)	UP (adj P Val<0.05), FC>1.5	Down (adj P Val<0.05)	Down (adj P Val<0.05), FC<-1.5
ATA+ *vs.* HC	4,372	2,393	215	1,979	238
ATA+(b) *vs.* HC	145	60	0	85	0
ATA+(a) *vs.* HC	7,639	3,868	919	3,771	1,506
ATA+(a) *vs.* ATA+(b)	6,839	3,490	834	3,349	1,393
ACA+ *vs.* HC	1	1	1	0	0
ARA+ *vs.* HC	0	0	0	0	0
SLE *vs.* HC	0	0	0	0	0

Supplemental Table 4: Numbers of differentially expressed genes in transcriptomics (derivation cohort).

DEG: differentially expressed genes; UP: overexpressed DEG; DOWN: underexpressed DEG; LogFC: log Fold Change; ATA+: anti-topoisomerase-I positive patients; ACA+: anti-centromere positive patients; ARA+: anti-RNA polymerase-III positive patients; SLE: systemic lupus erythematosus patients; HC: healthy controls; ATA+(a): ATA+ group a; ATA+(b): ATA+ group b.

Supplemental Table 5: List of differentially expressed genes in transcriptomics (main cohort).

Please refer to the Excel table.

**Group**	**Sex**	**Age at inclusion (years)**	**Cutaneous subtype**	**Disease duration at inclusion (years)**	**mRSS at inclusion**	**Organs involvement**	**Immunosuppressant treatment**	**ATA+ group**
**ATA+**	M	57	dcSSc	6	5	ILD<br>Telangiectasias	MMF	ATA+(b)
	M	48	dcSSc	1	7	ILD<br>DU	MMF	ATA+(a)
	M	53	dcSSc	4	7	ILD<br>DU<br>Telangiectasias	MTX	ATA+(a)
	F	53	dcSSc	2	8	ILD<br>Telangiectasias	MMF	ATA+(a)
	F	68	dcSSc	12	2	ILD<br>Telangiectasias	None	ATA+(a)
	F	35	dcSSc	16	5	ILD<br>DU<br>Telangiectasias	MMF	ATA+(a)
	M	64	dcSSc	11	22	ILD<br>Telangiectasias	MPA	ATA+(b)
	F	51	dcSSc	3	19	ILD<br>DU	TCZ	ATA+(a)
	F	36	dcSSc	11	5	DU<br>Telangiectasias	None	ATA+(b)
	M	76	dcSSc	1	2	ILD<br>DU	MTX	ATA+(a)
	F	59	dcSSc	3	6	ILD<br>DU<br>Telangiectasias	TCZ	ATA+(a)
	F	64	dcSSc	11	7	ILD	MMF	ATA+(a)
	F	81	lcSSc	13	2	ILD<br>Telangiectasias	None	ATA+(a)
	M	75	dcSSc	5	7	ILD	MMF	ATA+(b)

	M	65	dcSSc	12	2	ILD<br>DU<br>Telangiectasias	None	ATA+(a)
	M	70	dcSSc	2	8	None<br>Telangiectasias	MMF	ATA+(b)
	M	53	dcSSc	2	11	ILD<br>Telangiectasias	MTX	ATA+(b)
	F	82	dcSSc	5	0	ILD<br>Telangiectasias	None	ATA+(b)
	F	54	dcSSc	33	2	None<br>Telangiectasias	None	ATA+(b)
	M	55	dcSSc	5	6	ILD<br>DU<br>Telangiectasias	MMF	ATA+(b)
	F	71	lcSSc	10	4	None	None	ATA+(b)
	F	57	lcSSc	12	4	Telangiectasias	None	ATA+(b)
	M	72	dcSSc	6	21	ILD<br>DU	TCZ<br>MTX	ATA+(b)
	M	65	dcSSc	15	2	DU	None	ATA+(b)
**ACA+**	F	57	lcSSc	3	0	Telangiectasias	None	
	F	44	lcSSc	4	0	None	None	
	F	65	lcSSc	3	4	ILD<br>Telangiectasias	None	
	M	41	lcSSc	0	4	DU<br>Telangiectasias	None	
	F	66	lcSSc	3	2	Telangiectasias	None	
	F	67	lcSSc	1	4	Telangiectasias	None	
	F	31	lcSSc	2	0	Telangiectasias	None	
	F	64	lcSSc	7	3	Telangiectasias	MTX	
	F	76	lcSSc	2	5	Telangiectasias	None	
	F	70	lcSSc	6	5	Telangiectasias	None	
	F	76	lcSSc	6	2	None	None	
	F	62	lcSSc	0	2	Telangiectasias	None	

	F	40	lcSSc	0	2	Telangiectasias	None	
	F	56	lcSSc	0	2	None	None	
	F	46	lcSSc	0	0	Telangiectasias	None	
	M	61	lcSSc	4	10	DU<br>Telangiectasias	None	
	F	85	lcSSc	6	4	Telangiectasias	None	
	F	53	lcSSc	5	4	ILD<br>Telangiectasias	None	
**ARA+**	F	56	lcSSc	1	0	Telangiectasias	None	
	F	56	lcSSc	7	4	Telangiectasias	None	
	F	61	lcSSc	2	0	ILD<br>Telangiectasias	None	
	F	54	dcSSc	17	3	DU<br>Telangiectasias	MTX	
	F	78	dcSSc	5	18	ILD<br>Renal Crisis<br>Telangiectasias	MMF	
**HC**	F	31						
	F	36						
	F	40						
	F	54						
	F	54						
	F	54						
	F	56						
	F	62						
	F	64						
	F	64						
	F	66						
	F	67						
	F	68						
	F	69						

	M	41							
	M	48							
	M	53							
	M	61							
	M	65							
	M	70							

Supplemental Table 6: Subjects characteristics (validation cohort).

SSc: systemic sclerosis; dcSSc: diffuse cutaneous systemic sclerosis; lcSSc: limited cutaneous systemic sclerosis; ATA+: anti-topoisomerase-I positive patients; ATA+(a): ATA+ group a; ATA+(b): ATA+ group b; ACA+: anti-centromere positive patients; ARA+: anti-RNA polymerase-III positive patients; SLE: systemic lupus erythematosus patients; HC: healthy controls; ENA: extractable nuclear antigen; mRSS: modified Rodnan skin score; ILD: interstitial lung disease; PAH: pulmonary arterial hypertension; DU: digital ulceration; MMF: mycophenolate mofetil; MTX: methotrexate; MPA: mycophenolic acid; TCZ: tocilizumab.

Comparison	Total of DEP	UP (adj P Val<0.05)	UP (adj P Val<0.05), logFC>1	Down (adj P Val<0.05)	Down (adj P Val<0.05), logFC<-1
ATA+ *vs.* HC	403	290	23	113	16
ATA+(b) *vs.* HC	255	136	31	119	15
ATA+(a) *vs.* HC	732	475	35	257	44
ATA+(a) *vs.* ATA+(b)	903	515	19	388	50
ACA+ *vs.* HC	149	101	15	48	7
ARA+ *vs.* HC	0	0	0	0	0

Supplemental Table 7: Numbers of differentially expressed proteins in proteomics (validation cohort).

DEP: differentially expressed proteins; UP: overexpressed DEP; DOWN: underexpressed DEP; LogFC: log Fold Change; ATA+: anti-topoisomerase-I positive patients; ACA+: anti-centromere positive patients; ARA+: anti-RNA polymerase-III positive patients, ATA+(a): ATA+ group a; ATA+(b): ATA+ group b HC: healthy controls.

Supplemental Table 8: List of differentially expressed genes in proteomics (validation cohort).

Please refer to the Excel table.

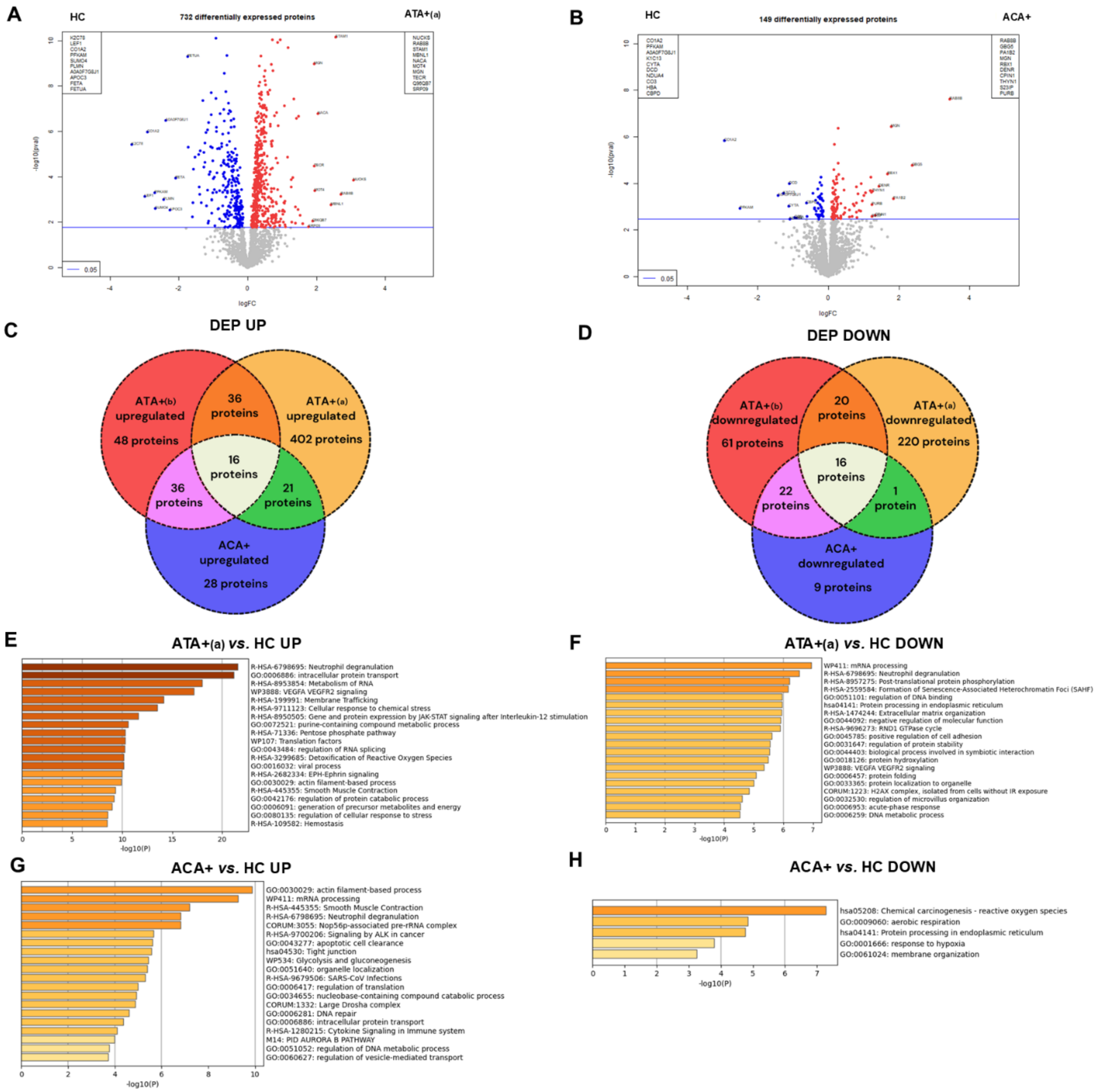
A
HC
732 differentially expressed proteins
ATA+(a)
logFC
-log10(pval)
0.05
B
HC
149 differentially expressed proteins
ACA+
logFC
-log10(pval)
0.05
C
DEP UP
ATA+(b) upregulated 48 proteins
36 proteins
ATA+(a) upregulated 402 proteins
16 proteins
36 proteins
21 proteins
ACA+ upregulated 28 proteins
D
DEP DOWN
ATA+(b) downregulated 61 proteins
20 proteins
ATA+(a) downregulated 220 proteins
16 proteins
22 proteins
1 protein
ACA+ downregulated 9 proteins
E
ATA+(a) vs. HC UP
R-HSA-6798695: Neutrophil degranulation
GO:0006886: intracellular protein transport
R-HSA-8953854: Metabolism of RNA
WP3888: VEGFA VEGFR2 signaling
R-HSA-199991: Membrane Trafficking
R-HSA-9711123: Cellular response to chemical stress
R-HSA-8950505: Gene and protein expression by JAK-STAT signaling after Interleukin-12 stimulation
GO:0072521: purine-containing compound metabolic process
R-HSA-71336: Pentose phosphate pathway
WP107: Translation factors
GO:0043484: regulation of RNA splicing
R-HSA-3299685: Detoxification of Reactive Oxygen Species
GO:0016032: viral process
R-HSA-2682334: EPH-Ephrin signaling
GO:0030029: actin filament-based process
R-HSA-445355: Smooth Muscle Contraction
GO:0042176: regulation of protein catabolic process
GO:0006091: generation of precursor metabolites and energy
GO:0080135: regulation of cellular response to stress
R-HSA-109582: Hemostasis
-log10(P)
F
ATA+(a) vs. HC DOWN
WP411: mRNA processing
R-HSA-6798695: Neutrophil degranulation
R-HSA-8957275: Post-translational protein phosphorylation
R-HSA-2559584: Formation of Senescence-Associated Heterochromatin Foci (SAHF)
GO:0051101: regulation of DNA binding
hsa04141: Protein processing in endoplasmic reticulum
R-HSA-1474244: Extracellular matrix organization
GO:0044092: negative regulation of molecular function
R-HSA-9696273: RND1 GTPase cycle
GO:0045785: positive regulation of cell adhesion
GO:0031647: regulation of protein stability
GO:0044403: biological process involved in symbiotic interaction
GO:0018126: protein hydroxylation
WP3888: VEGFA VEGFR2 signaling
GO:0006457: protein folding
GO:0033365: protein localization to organelle
CORUM:1223: H2AX complex, isolated from cells without IR exposure
GO:0032530: regulation of microvillus organization
GO:0006953: acute-phase response
GO:0006259: DNA metabolic process
-log10(P)
G
ACA+ vs. HC UP
GO:0030029: actin filament-based process
WP411: mRNA processing
R-HSA-445355: Smooth Muscle Contraction
R-HSA-6798695: Neutrophil degranulation
CORUM:3055: Nop56p-associated pre-rRNA complex
R-HSA-9700206: Signaling by ALK in cancer
GO:0043277: apoptotic cell clearance
hsa04530: Tight junction
WP534: Glycolysis and gluconeogenesis
GO:0051640: organelle localization
R-HSA-9679506: SARS-CoV Infections
GO:0006417: regulation of translation
GO:0034655: nucleobase-containing compound catabolic process
CORUM:1332: Large Drosha complex
GO:0006281: DNA repair
GO:0006886: intracellular protein transport
R-HSA-1280215: Cytokine Signaling in Immune system
M14: PID AURORA B PATHWAY
GO:0051052: regulation of DNA metabolic process
GO:0060627: regulation of vesicle-mediated transport
-log10(P)
H
ACA+ vs. HC DOWN
hsa05208: Chemical carcinogenesis - reactive oxygen species
GO:0009060: aerobic respiration
hsa04141: Protein processing in endoplasmic reticulum
GO:0001666: response to hypoxia
GO:0061024: membrane organization
-log10(P)

Supplemental Figure 2: Differential analyses in validation cohort.

Volcanoplot represents differential analysis between (A) ATA+(a) *vs.* HC and (B) ACA+ *vs.* HC. (C) Venn diagrams represent upregulated and (D) downregulated DEP in comparisons ATA+(b) *vs.* HC, ATA+(a) *vs.* HC and ACA+ *vs.* HC. (E) Enrichment analysis in upregulated DEP and (F) downregulated DEP in ATA+(a) group. (G) Enrichment analysis in upregulated DEP and (H) downregulated DEP in ACA+ group.

ATA+: anti-topoisomerase-I positive patients; ATA+(a): ATA+ group a; ATA+(b): ATA+ group b; ACA+: anti-centromere positive patients; HC: healthy controls; DEP: differentially expressed proteins.

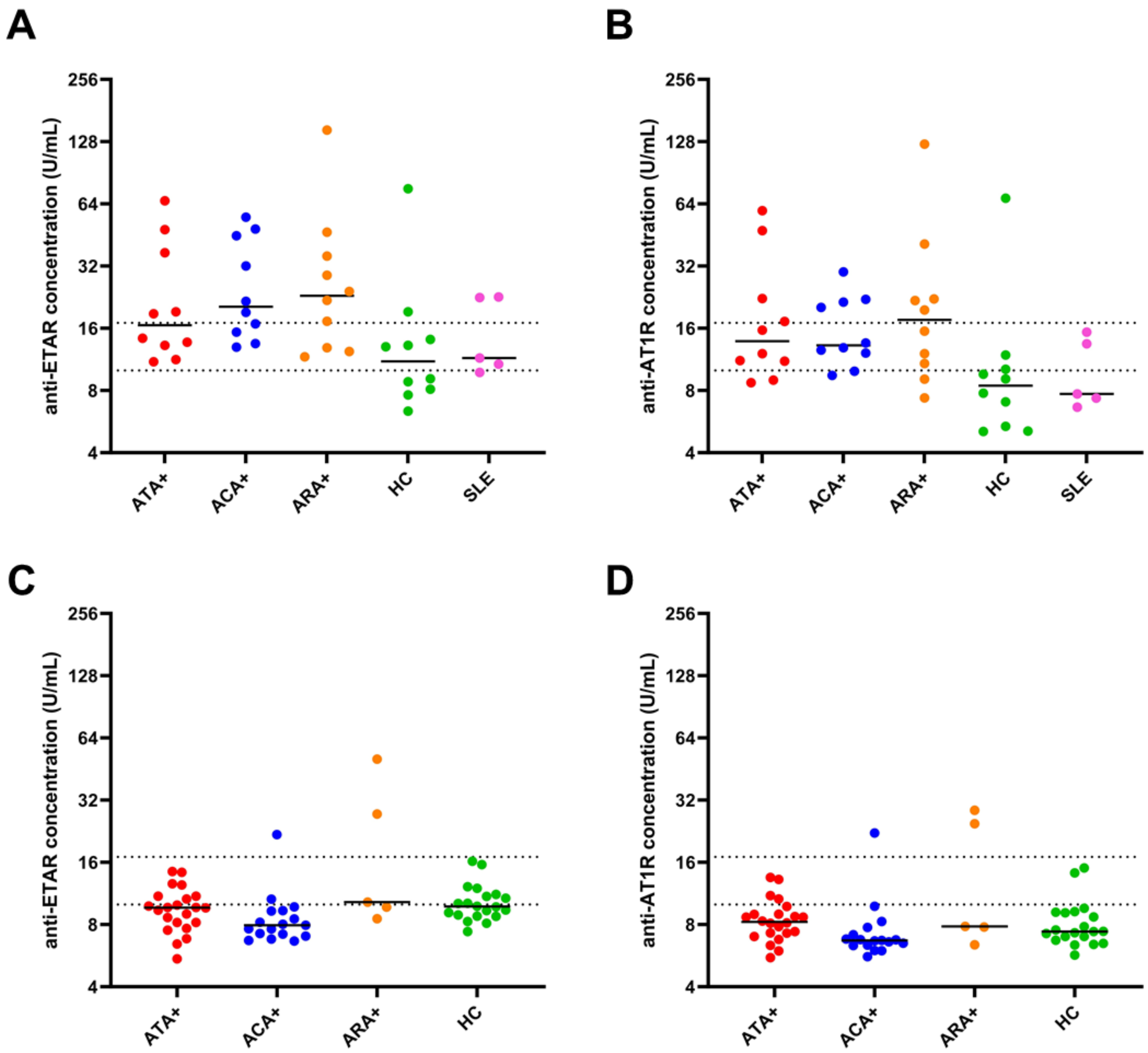

A
B
C
D
anti-ETAR concentration (U/mL)
anti-AT1R concentration (U/mL)
256
128
64
32
16
8
4
ATA+
ACA+
ARA+
HC
SLE

Supplemental Figure 3: Functionals antibodies levels.

ETAR (A) and AT1R (B) antibodies levels among groups derived from the cohort and ETAR (C) and AT1R (D) antibodies levels among group in validation cohort.

ATA+: anti-topoisomerase-I positive patients; ACA+: anti-centromere positive patients; ARA+: anti-RNA polymerase-III positive patients, SLE: systemic lupus erythematosus patients; HC: healthy controls, AT1R: angiotensin II type 1 receptor; ETAR: endothelin-1 type A receptor.

Protein name	Gene name
Signal Transducing Adaptor Molecule	STAM
Serine/threonine-protein kinase 24	STK24
Desmosome associated protein	PNN
Eleven proteins such as integrin alpha-3	ITGA3
VE-Cadherin	CDH5
Adaptor Subunit Of SYVN1 Ubiquitin Ligase	SEL1L
Rae1 ribonucleic acid export 1	RAE1
Epidermal growth factor receptor pathway substrate 15	EPS15
Nuclear receptor-binding protein	NRBP1
Cadherin 2	CDH2
Tripeptidyl-peptidase 1	TPP1
Intercellular Adhesion Molecule 1	I-CAM-1
Titin	TTN
Eukaryotic translation initiation factor 2B subunit gamma	EIF2B3
CDGSH iron sulfur domain 1	CISD1
Small Nuclear Ribonucleoprotein Polypeptide A	SNRPA
FKBP prolyl isomerase 11	FKBP11
Prolyl 4-hydroxylase subunit alpha-2	P4HA2

Supplemental Table 9: Targeted proteomic on validation cohort.

Panels of proteins based on derivation cohort proteomics and literature data were quantified by mass spectrometry based Absolute Quantification.

A

B

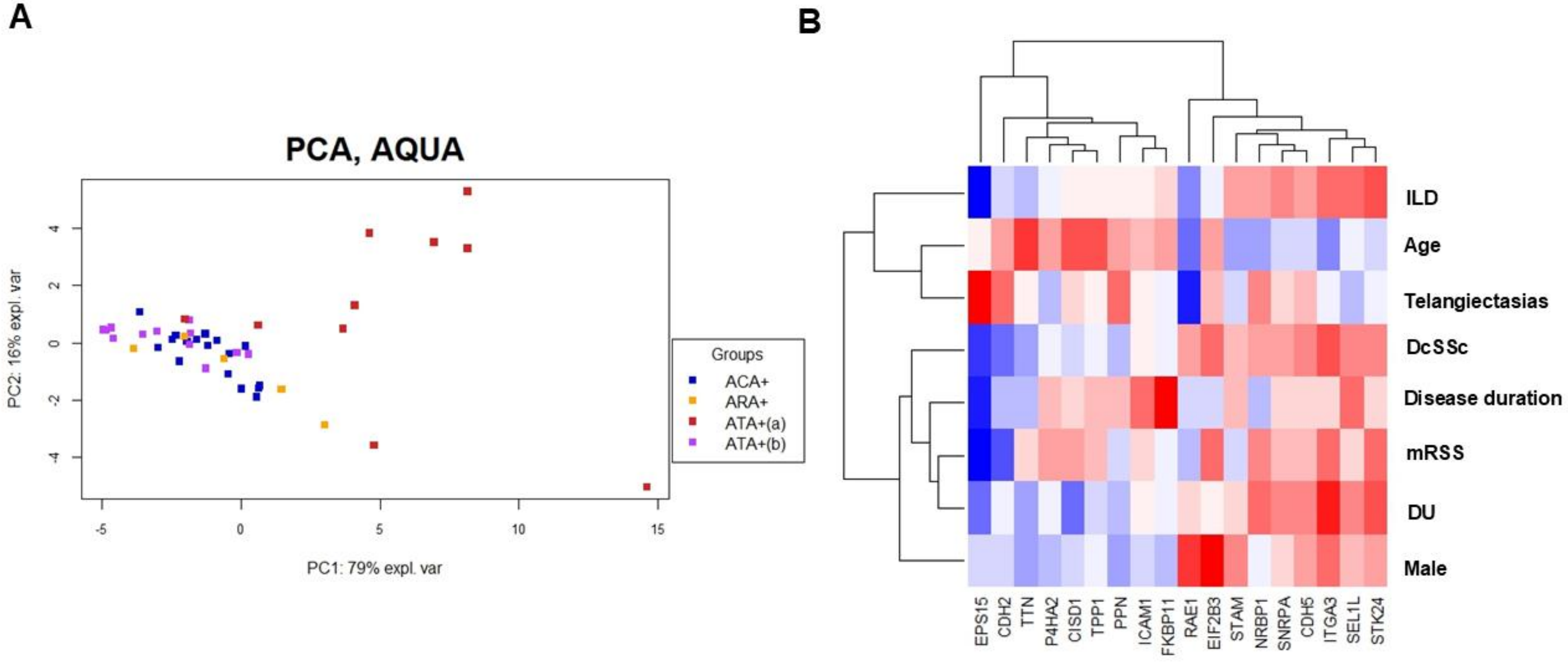

PCA, AQUA
PC2: 16% expl. var
PC1: 79% expl. var
Groups
ACA+
ARA+
ATA+(a)
ATA+(b)
ILD
Age
Telangiectasias
DcSSc
Disease duration
mRSS
DU
Male
EPS15
CDH2
TTN
P4HA2
CISD1
TPP1
PPN
ICAM1
FKBP11
RAE1
EIF2B3
STAM
NRBP1
SNRPA
CDH5
ITGA3
SEL1L
STK24

Supplemental Figure 4: Visualization of targeted proteomics performed by AQUA of validation cohort.

PCA scatter plots for the analyzed cell samples-based on proteins quantification by AQUA and clinical data (A). Heatmap represents correlations between proteins quantification by AQUA and clinical data (B).

ATA+: anti-topoisomerase-I positive patients; ACA+: anti-centromere positive patients; ARA+: anti-RNA polymerase-III positive patients. ATA+(a): ATA+ group a; ATA+(b): ATA group b; PCA: principal component analysis; AQUA: Absolute quantification proteomics.

CDH5: VE-Cadherin; EIF2B3: Eukaryotic translation initiation factor 2B subunit gamma; ITGA3: integrin alpha-3; NRBP1: Nuclear receptor-binding protein; P4HA2: Prolyl 4-hydroxylase subunit alpha-2; SEL1L: Adaptor Subunit Of SYVN1 Ubiquitin Ligase; SNRPA: Small Nuclear Ribonucleoprotein Polypeptide A; STAM: Signal Transducing Adaptor Molecule; STK24: Serine/threonine-protein kinase 24.

Supplemental Table 10: Detailed sequences and peptides sequences of targeted proteomic on the validation cohort

Please refer to the Excel table.

Panels of proteins based on derivation cohort proteomics and literature data were quantified by mass spectrometry based Absolute Quantification.
